# Changes in blood parameters of broilers fed solid-state fermented cassava peel–foliage mix meal as a replacement for *Zea mays* in broilers’ diets

**DOI:** 10.5455/javar.2025.l991

**Published:** 2025-12-25

**Authors:** Razaq Adekunle Animashahun, Olayinka Olubunmi Alabi, Adedeji Peculiar Animashahun, Olasunkanmi Peter Olajide, Abiodun Adebayo Idowu, Destiny Emmanuel Solomon, Oluwagbenga Paul Olorunfemi, Feranmi Gbenga Omoniyi, Collins Collins Francis, Emmanuel Oluwatobi Olowoloba

**Affiliations:** 1 Department of Animal Science, College of Agriculture, Landmark University, Omu-Aran, Nigeria; 2 Department of Animal Breeding and Genetics, Federal University of Agriculture, Abeokuta, Abeokuta, Nigeria; 3 Department of Food and Nutritional Sciences, University of Reading, Whiteknights Campus, Reading, United Kingdom

**Keywords:** Alternative feed, biochemical param, broiler nutrition, cassava by-products, hematological indices, sustainable feed alternatives

## Abstract

**Objective:**

The current study examined the effects of replacing *Zea mays* (maize) with solid-state Fermented cassava peel–foliage mix meal (FCPL) on the biochemical profiles and hematological markers.

**Materials and Methods:**

Cassava peels and foliage were processed by drying, grinding, and mixing at a ratio of 19:1; the resulting mixture was then fermented using *Aspergillus niger* American Type Culture Collection 16404. Then, broiler diets were supplemented with fermented cassava peel–­foliage mix meal (FCPL) at 0%, 20%, 40%, and 60% maize replacement levels. In a fully randomized design, 480 seven-day-old Anak 2,000 broiler chicks were assigned to the four dietary treatments. Each treatment included 120 birds, which were then split into four duplicates of 30 chicks each. **Results:** Inclusion of FCPL tended to improve hematological parameters, with hematocrit (PCV) increasing significantly (*p* < 0.05) and peaking at 60% replacement, while erythrocyte count and hemoglobin concentration showed numerical increases. Aspartate aminotransferase (AST) and alkaline phosphatase (ALP) levels were lower in FCPL-fed groups, suggesting no negative effects on liver function, whereas serum cholesterol and glucose levels reduced significantly (*p* < 0.05) as FCPL inclusion increased. Total serum protein remained within normal physiological ranges, and albumin concentration was highest at 40% replacement, suggesting optimal protein utilization at this level.

**Conclusion:**

Replacing maize with up to 60% FCPL in broiler diets enhances PCV levels, reduces serum cholesterol, and supports liver function. These findings highlight fermented cassava by-products as a sustainable, health-promoting, and cost-effective alternative energy source in poultry nutrition, contributing to feed resource diversification and improved productivity.

## Introduction

The global shortage of cereals has emerged as a critical issue, rendering the application of cereal-based feedstuffs economically unsustainable, particularly in developing regions. Monogastric animals, such as poultry, are especially vulnerable to this trend. About 70%–85% of the overall production expenditures in chicken farming are related to feed [1], posing a major barrier to profitability and discouraging new investment in the sector [2]. As a result, there is a pressing need to find alternate, locally obtainable, and profitable protein sources for broilers’ feed. Evaluating unconventional feed ingredients is essential to diversifying the resource base of poultry production [3].

Simultaneously, the swift expansion of the agricultural area has led to the generation of vast amounts of agro-­industrial residues and byproducts. Due to the production of greenhouse gases and potential contamination of soil and water resources, the improper disposal of these materials, typically through burning or landfilling, poses significant risks to the environment and human health [4–6]. These residues, predominantly lignocellulosic in nature, are generally low in nutritional value and contain anti-nutritional factors, making them unsuitable for direct use in animal feed [7].

One such underutilized agro-industrial residue is the peel from cassava, a byproduct that accounts for approximately 15% of the cassava root [8,9]. Due to their low nutritional profile and the presence of anti-nutritional compounds, cassava peels are rarely used in poultry diets, despite their abundance, resulting in significant waste and environmental degradation [10]. Similarly, cassava foliage, rich in protein, minerals, and carotenoids, offers potential as a feed resource, but its use is limited by high hydrocyanic acid (HCN) levels, low energy density, and bulkiness [11]. Solid-state fermentation (SSF) has been suggested as a sustainable biotechnological strategy to improve the nutritional value of agro-industrial residues, thereby overcoming these restrictions [12,13]. SSF employs specific microorganisms, primarily filamentous fungi such as *Aspergillus*, *Rhizopus*, and *Penicillium*, to BioConvert complex substrates under low-moisture conditions [14]. Compared to submerged fermentation, SSF is more energy-efficient and environmentally friendly, aligning with the tenets of the circular economy [15]. SSF has shown promise in improving the nutritional value of cassava peel by reducing anti-nutritional factors and breaking down complex lignocellulosic structures.

However, before such fermented products can be integrated into poultry diets, their effects on animal health must be rigorously assessed. Blood and biochemical traits are key indicators of functional and nutritional status, providing insight into the safety and efficacy of novel feed ingredients [16,17]. Past studies have reported mixed results, with some indicating improvements in white blood cell counts and serum metabolites following the inclusion of fermented cassava in poultry diets [16,18] Others report no significant effects on red blood cell parameters.

Building on this context, the current study aims to assess the impact of replacing *Zea mays* (maize) with SSF cassava peel-foliage mix on the hematological and serum biochemical profile of broilers. Specifically, it seeks to assess the hematological and biochemical responses of birds to this alternative feed formulation, thereby contributing to efforts in sustainable poultry nutrition and waste valorization.

## Materials and Methods

### Ethical approval

With special attention to guaranteeing the health, welfare, and humane treatment of the broilers throughout the trial period, the experiment was conducted strictly in accordance with internationally recognized norms governing the handling and use of animals in experimental investigations. The Landmark University Research and Ethics Committee granted ethical approval for the research protocol, and the study was conducted under Certificate No. LMUIREC/ACSC/041/2025.

### Study site and period

From the final 2 weeks of March to the 4th week of April 2025, a 6-week feed trial experiment was carried out at the Teaching and Research Farm (Poultry Unit), Landmark University, Omu-Aran, Kwara State, Nigeria. The study site is located at 8°09ʹN latitude, 5°61ʹE longitude, and 564 m above sea level.

### Ingredient sources

Cassava peels and foliage (leaves) were sourced from the University cassava mill, while other feed resources were obtained from Omu-Aran, Kwara State.

### The organism’s source

The Microbiology Department at Landmark University provided *A*. *niger* American Type Culture Collection (ATCC) 16404, which was cultivated on potato dextrose agar at 25°C for seven days. Using a hemocytometer (Fuchs-Rosenthal method), spores were collected by tapping inverted plates, and the results showed 1.07 × 10⁹ sfu/ml.

### Preparation of test ingredients

Cassava peels and foliage were air-dried in an aerated environment until the leaves became brittle without losing color and the peels developed a crisp texture. The dried materials were then homogenized at a ratio of 19:1 (19 kg of peels to 1 kg of leaves) to obtain the cassava peel–leaf mix.

### Fermentation procedure

A cassava peel–leaf mix meal (2 kg) was hydrated with distilled water (1:1), sterilized (121°C, 103.421 kPa, 15 min), cooled, and then transferred to sterilized trays (58 × 38 × 4 cm) lined with cellophane. Substrates were inoculated under laminar airflow with 200 ml of *A*. *niger* ATCC 16404 (1.07 × 10⁹ sfu/ml) and incubated at ambient temperature for 96 h, then air-dried (~10% moisture) and stored in cellophane bags for feed incorporation.

### Proximate and anti-nutritional factors

The Official Methods of Analysis of AOAC International [19] standard techniques were used to determine the proximate composition, which included moisture, crude protein, Ether extract, crude fiber, ash, and nitrogen-free extract. Hydrogen cyanide content was analyzed using AOAC Method 915.03, phytate using AOAC Method 986.11-1988, and tannin using AOAC Method 952.03. The flavonoid and saponin contents were quantified according to the procedures described by Achikanu and Ani [20].

### Experimental design and management of broilers

In a fully randomized design, 480 Anak 2,000 broiler chicks (1 week old) were divided into four feeding regimens (120 chicks per treatment, with 4 replicates of 30 birds each) based on their average initial weight. After being raised on deep litter for 7 weeks, the birds were acclimated to a commercial starter food for 7 days before beginning experimental feeding. Regular vaccinations, medications, and husbandry were carried out in accordance with Ag Guide requirements, and food and water were given freely [21].

### Experimental feeds

For both the starter and finisher phases, four diets were created by substituting fermented cassava peel–foliage mix meal (FCPL) for maize at weight-for-weight inclusion levels of 0% (control), 20%, 40%, and 60%, while keeping the other ingredients unchanged (Table 3). Crude protein content ranged from 23.90% (control) to 23.19% (60% FCPL) in starter diets and from 20.72% to 19.76% in finisher diets. Corresponding metabolizable energy values were 3,036–2,935 kcal/kg (starter) and 3,073–2,953 kcal/kg (finisher).

**Table 3.** Composition (%) of the diets fed to the experimental diets (on a dry matter basis).

Ingredients (%)	Starter’s phase				Finisher’s phase			
	Diet 1	Diet 2	Diet 3	Diet 4	Diet 1	Diet 2	Diet 3	Diet 4
Corn	56.00	44.80	33.60	22.40	65.00	52.00	39.00	26.00
FCPL	0.00	11.20	22.40	33.60	0.00	13.00	26.00	39.00
Fish meal	2.00	2.00	2.00	2.00	1.20	1.20	1.20	1.20
SBM	38.10	38.10	38.10	38.10	30.00	30.00	30.00	30.00
Methionine	0.25	0.25	0.25	0.25	0.20	0.20	0.20	0.20
Bone meal	3.00	3.00	3.00	3.00	3.00	3.00	3.00	3.00
Salt	0.25	0.25	0.25	0.25	0.25	0.25	0.25	0.25
Lysine	0.15	0.15	0.15	0.15	0.10	0.10	0.10	0.10
Premix	0.25	0.25	0.25	0.25	0.25	0.25	0.25	0.25
Toxin binder	0.10	0.10	0.10	0.10	0.00	0.00	0.00	0.00
Total	100	100	100	100	100	100	100	100
Calculated analysis								
CP (%)	23.90	23.66	23.45	23.19	20.72	20.40	20.08	19.76
ME	3036	2948	2969	2935	3073	3031	2993	2953

CP = Crude protein; D1 = Control diet without FCPL; D2 = Diet containing 20% FCPL; D3 = Diet containing 40% FCPL; D4 = Diet containing 60% FCPL; FCPL = Fermented cassava peel-foliage mix meal, ME (kcal/kg) = Metabolizable energy.

### Blood collection for hematological and biochemical studies

Three overnight-fasted chickens per replication had their brachial veins drawn for blood samples (12 samples/treatment). Samples were placed in plain tubes for biochemistry analysis and ethylenediaminetetraacetic acid-coated tubes for hematology. After allowing the plain samples to clot for 6 h at 25°C and centrifuging them for 4 min at 2,000 rpm, the serum was decanted and stored at −20°C. Hematological parameters were determined using a Sysmex K-1000 hematology analyzer (Sysmex Corp., Kobe, Japan), while biochemical indices were assayed with commercial kits (Sigma Co., St. Louis, MO). Hemoglobin (Hb), hematocrit (PCV), and erythrocyte (RBC) counts were used to calculate RBC indices.


$$\mathrm{MCV}(\mu 3)=\frac{\mathrm{PCV}}{\mathrm{RBC}}\times 10$$

$$\mathrm{MCH}(\mathrm{pg})=\frac{\mathrm{Hb}(\mathrm{gm}/100\mathrm{ml})}{\mathrm{RBC}}\times 10$$

$$\mathrm{MCHC}(\mathrm{gm}/\mathrm{dl})=\frac{\mathrm{Hb}(\mathrm{gm}/100\mathrm{ml})}{\mathrm{PCV}}\times 10$$

### Statistical analysis

Hematological and serum biochemical parameter data were represented as averages of three replicates per treatment and subjected to ANOVA using SPSS’s General Linear Model technique. The statistical model used was: Y_ij_ = μ + T_i_ + e_ij_.

Observations (Y_ij_) were modeled as the overall mean (μ), treatment effect (T_i_), and random error (e_ij_); treatment means were separated by Duncan’s Multiple Range Test and expressed as mean ± SEM, with significance set at *p* < 0.05.

## Results and Discussion

Table 1 shows the proximate composition of fermented cassava peel–leaf meal (moisture 12.96 %, crude protein 7.83 %, crude fiber 10.34 %, ether extract 11.95 %, ash 8.33 %, and nitrogen-free extract 48.59 %), indicating that SSF enhanced its nutritional quality. It demonstrated the SSF capacity to improve the nutrient quality of the cassava peel-foliage meal. The impact of fermentative bacteria is reflected in the moderate crude protein value, which corroborates studies showing the protein enhancement capacity of SSF on agro-waste [22]. The proportionately high ether extract value signifies a potential source of additional dietary energy, while the ash content shows a good potential mineral source. Although the carbohydrate fraction is lower than in cereals, it still provides a substantial energy base. Compared to maize and soybean meals, FCPL is limited in protein but higher in fiber; yet, fermentation helps close this gap. In practice, it can serve as an energy- and mineral-rich supplement alongside protein-rich ingredients. A recent meta-analysis confirms that fermented cassava by-products can maintain broiler performance when included at controlled levels [23].

**Table 1.** Proximate components of the fermented cassava peel-foliage meal.

Param (%)	
Moisture	12.96
Crude protein	7.83
Crude fibre	10.34
Ether extract	11.95
Ash	8.33
Nitrogen-free extract	48.59
Total	100.00

### Anti-nutritional factors screening of fermented cassava peel-foliage meal

Table 2 indicates the anti-nutritional profile (hydrocyanide 1.03 mg/kg, phytate 10.91 mg/100 gm, alkaloid 3.09 %, saponin 1.88 %, and tannin 0.34 %) shows that SSF effectively reduced toxic factors. The very low cyanide content reflects enzymatic hydrolysis of cyanogenic glycosides and volatilization of HCN, as observed in other fermented cassava products [24]. Phytate levels remained moderate but were likely degraded, at least in part, by microbial phytases, thereby improving mineral availability [25]. Alkaloid and saponin values were within safe limits for poultry and lower than many browse plants, while tannin was minimal and far below levels in sorghum or legumes that hinder nutrient utilization. These results align with findings that fermented cassava leaf meal improves broiler growth, carcass traits, and nutrient digestibility [26], while mixtures with *Moringa oleifera* enhance growth and antioxidant status [27]. Broader reviews confirm that fermentation reduces anti-nutrients while generating organic acids, enzymes, and metabolites that support gut health and nutrient absorption [28].

**Table 2.** Antinutritional composition of the fermented cassava peel-foliage meal.

Anti-nutritional factors	
Hydrocyanide (mg/kg)	1.03
Phytate (mg/100 gm)	10.91
Alkaloid (%)	3.09
Saponin (%)	1.88
Tannin (%)	0.34

### The SSF cassava-based diets’ effect on hematological indices

The effect of the fermented cassava by-product-based diet on the hematological profile of the broilers is shown in Table 4. The RBC, the Hb, and the WBC were enhanced by the Fermented cassava peel–foliage mix meal, but the PCV was significantly impacted (*p* < 0.05). The apparent rise in RBC (3.30 × 10⁶/ml), Hb (12.03 gm/dl), and PCV (32.37%) at 60% replacement is an indication of elevated red blood cell production and blood oxygen levels, which improve the physiology of the broilers. The WBC was highest at 40% FCPL (12.53 × 10³/ml), but lower at 60%, demonstrating a boosted immune response at lower levels and possible suppression at higher inclusion. The RBC report corresponds to that of Sanusi et al. [29], which is higher than those of Aro et al. [30] and Sugiharto et al. [31]. Notably, it falls within the reported normal range by Jain [32], indicating the safe use of FCPL.

**Table 4.** Hematological indices of 480 broilers fed diets containing fermented cassava by-products.

Param	Level of fermented cassava peel-foliage mix meal				± SEM
	Diet 1 (0%)	Diet 2 (20%)	Diet 3 (40%)	Diet 4 (60%)	
RBC (×10^6^/ml)	3.14	3.17	3.30	3.30	0.34
Hematocrit (%)	30.60^b^	31.50^b^	32.03^a^	32.37^a^	2.15
Hemoglobin (gm/dl)	11.20	11.53	11.93	12.03	0.29
MCV (fl)	97.45	99.37	97.06	98.09	0.29
MCH (pg)	35.67	36.37	36.15	36.45	0.38
MCHC (%)	36.60	36.60	37.25	37.16	0.45
WBC (×10^3^/ml)	11.78	12.18	12.53	11.60	0.53
Platelets (×10^3^/ml)	3.33	3.26	3.30	3.37	0.25

^a,b^Means with different superscripts between the replacement levels are significantly different (*p* < 0.05); MCH = Mean corpuscular haemoglobin; MCHC = Mean corpuscular hemoglobin concentration; MCV = Mean corpuscular volume; RBC = Erythrocytes count; WBC = Leucocytes count.

The beneficial increases and elevations in hematological indices demonstrate the capacity of the FCPL to enhance red blood cell production and increase hemoglobin concentration. However, only the PCV was significantly different (*p* < 0.05) and exceeded that of Aguihe et al. [33], while the others showed a promising pattern; therefore, their description should be cautious. Hence, there is a need for further research to authenticate these findings. The blending of cassava foliage (leaves) into the peel by fermentation enriches the diet with vitamins and important minerals [34], which improve the process of blood production in the broilers while the parameters remain within normal limits for PCV and hemoglobin (5.9%–41.0%; 11.60%–13.68%) in the birds [35,36].

This study recorded a higher mean corpuscular volume (macrocytosis) and mean corpuscular hemoglobin but returned a lower mean corpuscular hemoglobin concentration than the report by Wikivet [35], which is comparable to the work by Ehebha and Eguaoje [37] on broilers fed sundried cassava peel-based diets fortified with an exogenous enzyme (Maxigrain). Macrocytosis is associated with deficiencies of vitamin B12 and folic acid, as well as an enlargement of the RBC [38]. The leucocyte, a WBC differential, aligns with the reported range by Chidi et al. [39] but is lower than that of Duwa et al. [40], demonstrating physiological stability under reduced immune stress. The WBC count determines the level of immunity [41]; it was normal across the treatments, indicating that FCPL did not cause immune perturbations but rather enhances leukocyte immunological balance in broilers in the current study [42].

The hematological indices observed in this study, except for WBC count, were consistent with those reported by Adeyemo et al. [43] in broilers fed *A*.* niger*–hydrolyzed cassava peel diets.

### Changes in the serum biochemical indices

The biochemical indices were significantly influenced (*p* < 0.05) by corn replacement with FCPL (Table 5), consistent with reports that diet strongly modulates serum biochemistry in broiler chickens [44]. Alkaline phosphatase (ALP), aspartate aminotransferase (AST), and alanine aminotransferase (ALT) are critical biomarkers of liver and organ function in broiler studies, and shifts in their activities provide insights into the metabolic effects of alternative feeds such as cassava by-products [45]. In the present study, ALT activity increased with rising FCPL inclusion, whereas ALP and AST levels declined progressively. The highest ALP and AST activities were observed in broilers fed the control diet. The AST and ALT levels recorded here exceeded those reported by Aro et al. [37]. The AST range was lower than that of Sugiharto et al. [31], with such variations attributable to differences in diet, growth stage, stress, genetics, disease status, or liver and muscle physiology. The higher crude protein and metabolizable energy contents of the diets used in this study relative to those of Aro and Aletor [46] and Sugiharto et al. [31] may explain the elevated transaminase levels, consistent with Bona et al. [47], who observed increased ALT, AST, and AST: ALT ratios in broilers on high-protein diets. Elevated transaminases are typically linked to hepatic or muscular damage, and the susceptibility of fast-growing broilers to muscle injury may underline the higher AST:ALT ratios [47]. Impaired liver function can further contribute to the plasma accumulation of ammonia and increased hepatic enzyme activities [48]. Additionally, associations between altered liver enzymes and cognitive outcomes have also been reported in humans with fatty liver disease [49]. The ALP values were within acceptable ranges, as reported by Jain [32], indicating that the FCPL did not impair metabolic activities. Although ALP elevation typically signals disruption of the bile duct [50,51, this was not observed in our study.

**Table 5.** Serum biochemical indices of 480 broilers fed diets containing fermented cassava by-products.

Param	Level of fermented cassava peel- leaf mix meal				± SEM
	Diet 1 (0%)	Diet 2 (20%)	Diet 3 (40%)	Diet 4 (60%)	
ALT (IU)	4.99^b^	4.77^b^	5.35^a^	5.47^a^	2.29
ALP (IU)	104.53^a^	99.33^b^	88.93^c^	82.65^cd^	2.50
AST (IU)	128.65^a^	126.79^a^	121.35^b^	121.68^b^	4.34
Glucose (mg/dl)	108.21^a^	105.10^a^	100.34^ab^	90.93^b^	1.26
TP (gm/dl)	5.90^ab^	6.00^a^	6.13^a^	5.49^b^	1.17
Albumin (gm/dl)	3.54^a^	3.56^a^	3.56^a^	3.19^b^	2.12
Globulin (gm/dl)	2.36^b^	2.44^ab^	2.57^a^	2.30^b^	1.20
CHO (mg/dl)	114.81^a^	108.45^ a^	97.91^b^	96.40^b^	3.39

^a–d^Means with different superscripts between the replacement levels are significantly different (*p* < 0.05); ALT = Alanine transaminase; ALP = Alkaline phosphatase; AST = Aspartate aminotransferase; CHO = Cholesterol; TB = Total protein.

The serum glucose and cholesterol concentrations decreased with the FCPL inclusion level, peaking at the 60% replacement level. However, these values were below the Aro et al. [30] report and above that of Abdulazeez et al. [52], which may imply FCPL’s capacity to induce hypoglycemia, an occurrence in some plant protein sources like the cassava leaf, and possibly from *A*.* niger*-linked fermented products like the lactic acid, which moderates glucose transportation [53,54].

The serum TP at 20% FCPL was comparable to that of the control diet birds, while the intermediate FCPL diet (40%) value was higher; it was generally high for all FCPL diets, demonstrating enhanced nutrient provision in the FCPL diets [55]. The high albumin at 40% replacement demonstrates the FCPL protein quality, as the albumin sustains a balanced osmosis, facilitates fluid distribution, and serves as a carrier for steroid hormones, hemin, and fatty acids [56], while globulins contribute to immunoglobulin synthesis, although abnormally high levels may result from medications, dehydration, infections, or immune-related disorders [57].

As the replacement level of FCPL increased, the serum cholesterol levels were inversely proportional, in consonance with Shuvo et al. [58] on broilers fed fermented rice bran and Ehebha and Eguaoje [37], but fell below the Aro et al. [30] report. This FCPL modulatory effect may be connected to the SSF process and its nutritional content.

The *A*.* niger* ATCC 16404 SSF-based process was able to break down hardy fibers and ANFs in the FCPL by-product [59], releasing bound nutrients, enhancing useful secondary metabolites, and releasing short-chain fatty acids that inhibit liver synthesis, such as butyrate. The fiber content from the cassava leaf and peel benefits the gastrointestinal tract (GIT) integrity and reduces cholesterol digestion in the GIT [60] and controls delivery to the liver by binding bile acids, improving excretion, and liver cholesterol intake and synthesis [61]. SSF generates enzymes that modulate the inflow of lipoproteins in circulation and their proliferation [53], while the secondary metabolites in cassava leaves, such as saponin, moderate gut absorption by exerting a further lipidemic effect [62]. The liver cholesterol synthesis is also reduced by the low calorie of the FCPL [63], apparently demonstrating the low serum cholesterol in the FCPL-fed birds [27] and within accepted limits [64]. These patterns showed that the FCPL did not compromise liver function, metabolic health, or nutritional status, thereby supporting its safety and efficacy as a sustainable maize substitute in broiler nutrition.

The study’s limitations included flock size, management, ethical considerations, and blood sampling size. However, further studies on a larger flock size are recommended to validate the outcomes of this study.

## Conclusion

The FCPL impact in this research has been proven beneficial to gut wellbeing, nutrient availability, and partitioning, without adverse effects on blood indices and liver functionality. Based on these findings, it can replace maize up to 60%, as it is locally available all year round and is cost-saving. FCPL is therefore a viable and sustainable feed ingredient that enhances broiler physiological status and supports profitable poultry production, warranting further investigation to optimize fermentation methods, assess long-term carcass quality, and evaluate the commercial-scale economic and environmental impacts.

## List of abbreviations

Ag, agricultural; ALP, alkaline phosphatase; ALT, alanine aminotransferase; ANOVA, analysis of variance; AST, aspartate aminotransferase; ATCC, American Type Culture Collection; C, Celsius; cm, centimeter; Co, company; CRD, completely randomized design; DMRT, Duncan’s multiple range test; E, east; FCPL, Fermented cassava peel–foliage mix meal; GLM, general linear model; gm/dl, gram per deciliter; Hb, hemoglobin; kg, kilogram; kPa, kilopascal; MCH, mean corpuscular hemoglobin; MCHC, mean corpuscular hemoglobin concentration; MCV, mean corpuscular volume; ml, milliliter; N, north; PCV, hematocrit; pg, picogram; RBC, red blood cell count; SFU, spore-forming units per milliliter; SPSS, Statistical Package for the Social Sciences; SSF, solid-state fermentation; TP, total protein; WBC, leucocyte count.

## References

[1] Wongnaa CA, Mbroh J, Mabe FN, Abokyi E, Debrah R, Dzaka E, et al. (2023). Profitability and choice of commercially prepared feed and farmers’ own prepared feed among poultry producers in Ghana. J Agric Food Res.

[2] Adams F, Mensah A, Etuah S, Aidoo R, Asante BO, Mensah JO (2022). Modelling of vertical integration in commercial poultry production of Ghana: a count data model analysis. Heliyon.

[3] Getiso A, Asrat M, Tesfaye E (2021). Assessing protein value of cassava (*Manihot esculenta* Crantz) leaf meal: effect on feed intake, growth performances and carcass characteristics of Potchefstrom Koekoek. J Anim Res Nutr.

[4] Adejumo IO, Adebiyi OA (2021). Agricultural solid wastes: causes, effects, and effective management. Strategies of sustainable solid waste management.

[5] Kolawole ID, Kolawole GO, Sanni-Manuel BA, Kolawole SK, Ewansiha JU, Kolawole VA, et al. (2024). Economic impact of waste from food, water, and agriculture in Nigeria: challenges, implications, and applications—a review. Discov Environ.

[6] Sadh PK, Duhan S, Duhan JS (2018). Agro-industrial wastes and their utilization using solid-state fermentation: a review. Bioresour Bioprocess.

[7] Yafetto L, Odamtten GT, Wiafe-Kwagyan M (2023). Valorization of agro-industrial wastes into animal feed through microbial fermentation: a review of the global and Ghanaian case. Heliyon.

[8] Abouelezz K, Yuan J, Wang G, Bian G (2018). The nutritive value of cassava starch extraction residue for growing ducks. Trop Anim Health Prod.

[9] Ogbuewu IP, Mbajiorgu CA (2023). Utilisation of cassava as a source of energy and protein in broiler chicken and laying hen diets. Trop Anim Health Prod.

[10] Chinedu I, Ezennia IS, Onuorah IM, Osita ES (2023). Effective waste management: a panacea for environmental pollution in cassava processing factories in Nigeria. Int J Innov Environ Stud Res.

[11] Chaiareekitwat S, Latif S, Mahayothee B, Khuwijitjaru P, Nagle M, Amawan S, et al. (2022). Protein composition, chlorophyll, carotenoids, and cyanide content of cassava leaves (*Manihot esculenta* Crantz) as influenced by cultivar, plant age, and leaf position. Food Chem.

[12] Farinas CS (2015). Developments in solid-state fermentation for the production of biomass-degrading enzymes for the bioenergy sector. Renew Sust Energ Rev.

[13] Yafetto L (2022). Application of solid-state fermentation by microbial biotechnology for bioprocessing of agro-industrial wastes from 1970 to 2020: a review and bibliometric analysis. Heliyon.

[14] Lizardi-Jiménez MA, Hernández-Martínez R (2017). Solid-state fermentation (SSF): diversity of applications to valorize waste and biomass. 3 Biotech.

[15] Ojo AO, De Smidt O (2023). Lactic acid: a comprehensive review of production to purification. Processes.

[16] Irekhore OT, Adeyemi OM, Idowu OMO, Akinola OS, Bello KO (2015). Growth performance, haematological indices and cost benefits of growing pigs fed cassava peel meal diets supplemented with Allzyme SSF. Int J Appl Agric Apic Res.

[17] Cruz CEB, Freitas ER, Braz N, Salles RPR, Da Silva ING (2018). Blood param and enzymatic and oxidative activity in the liver of chickens fed with calcium anacardate. Rev Cienc Agron.

[18] Ogbuewu IP, Mabelebele M, Mbajiorgu CA (2023). Meta-analysis of blood indices and production physiology of broiler chickens on dietary fermented cassava intervention. Trop Anim Health Prod.

[19] Latimer GW Jr, editor (2023). Official methods of analysis of AOAC International. AOAC Publications.

[20] Achikanu CE, Ani ON (2020). Nutritional and phytochemical content of *Cissus populnea* (Okoho) stem bark. Asian J Res Biochem.

[21] Salak-Johnson J (2020). The Ag guide serves as a primary standard for animal scientists and AAALAC accreditation of Ag research programs. J Anim Sci.

[22] Sousa D, Moset V, López-Luján MDC, Salgado JM, Dias A, Belo I, et al. (2024). Potential of solid-state fermentation to enhance the nutritional value of oilseed cakes for poultry. Anim Feed Sci Technol.

[23] Yang Y, Lei F, Zhang Z, Liu L, Li Q, Guo A (2024). Effects of cassava root meal on the growth performance, apparent nutrient digestibility, organ and intestinal indices, and slaughter performance of yellow-feathered broiler chickens. Trop Anim Health Prod.

[24] Dagaew G, Wongtangtintharn S, Prachumchai R, Cherdthong A (2023). The effects of fermented cassava pulp with yeast waste and different roughage-to-concentrate ratios on ruminal fermentation, nutrient digestibility, and milk production in lactating cows. Heliyon.

[25] Samtiya M, Aluko RE, Dhewa T (2020). Plant food anti-nutritional factors and their reduction strategies: an overview. Food Prod Process Nutr.

[26] Bhavna A, Zindove TJ, Iji PA, Bakare AG (2024). Growth performance, carcass characteristics and meat sensory evaluation of broiler chickens fed diets with fermented cassava leaves. Anim Biosci.

[27] Sugiharto S, Widiastuti E, Isroli I, Yudiarti T, Sartono TA, Wahyuni HI (2020). Effect of feeding fermented mixture of cassava pulp and *Moringa oleifera* leaf meal on immune responses, antioxidative status, biochemistry indices, and intestinal ecology of broilers. Vet World.

[28] Sugiharto S (2016). Role of nutraceuticals in gut health and growth performance of poultry. J Saudi Soc Agric Sci.

[29] Sanusi M, Rabi A, Dom UD, Haruna J (2015). Comparative effect of self-fix-case-formulated and four commercial diets on the growth performance, carcass and haematological param of broiler finishers in the tropics. Sokoto J Vet Sci.

[30] Aro SO, Agbede JO, Dairo OO, Ogunsote E, Aletor VA (2012). Evaluation of fermented cassava tuber wastes in broiler chickens feeding. Arch Zootech.

[31] Sugiharto S, Yudiarti T, Isroli I, Widiastuti E, Putra FD (2017). Effect of dietary supplementation with *Rhizopus oryzae* or *Chrysonilia crassa* on growth performance, blood profile, intestinal microbial population, and carcass traits in broilers exposed to heat stress. Archiv Für Tierzuchtung.

[32] Jain NC (1993). Educational Essentials of veterinary haematology. Educational Essentials of veterinary haematology.

[33] Aguihe PC, Kehinde AS, Abdulmumini S, Ospina-Rojas IC, Murakami AE (2017). Effect of dietary probiotic supplementation on carcass traits and haematological responses of broiler chickens fed shea butter cake based diets. Anim Sci.

[34] Sharma A, Sharma R, Sharma M, Kumar M, Barbhai MD, Lorenzo JM, et al. (2022). *Carica papaya* L. leaves: deciphering its antioxidant bioactives, biological activities, innovative products, and safety aspects. Oxid Med Cell Longev.

[35] WikiVet (2012). Chicken haematology. https://en.wikivet.net/Chicken_Haematology.

[36] Upah SO, Orayaga KT, Yakubu RN, Gyang IY, Odeh OM, Magaji ST (2024). Haematological and biochemical indices of finisher broiler chickens fed graded levels of spurge weeds (*Euphorbia heterophylla*) leaf meal. Open J Anim Sci.

[37] Ehebha ETE, Eguaoje AS (2018). Haematological and serum biochemical indices of broiler chickens fed varying dietary levels of sun-dried cassava (*Manihot esculenta*) peel meal supplemented with enzyme (Maxigrain®). Asian J Res Anim Vet Sci.

[38] Russell JE, Wilson M (2019). Anemia: low hemoglobin, low hematocrit, low red cell count. Cancer Ther Advisor.

[39] Chidi UL, Ebenebe CI, Ngozi OP (2024). Growth performance, haematological and serum biochemistry of broilers fed diets containing *Vitex doniana* leaf meal. Rev Cienc Agroveterinarias.

[40] Duwa H, Saleh B, Lamido M, Saidu A (2014). Growth, haematological and serum biochemical indices of broiler chickens fed banana peel meal as replacement for maize in the semi-arid zone of Nigeria. Online J Anim Feed Res.

[41] Mank V, Azhar W, Brown K (2024). Leukocytosis. In: StatPearls. StatPearls Publishing, Treasure Island, USA.

[42] Tigner A, Ibrahim S.A, Murray IV (2025). Histology, white blood cell. In: StatPearls.

[43] Adeyemo IA, Sani A (2013). Haematological param and serum biochemical indices of broiler chickens fed Aspergillus niger hydrolyzed cassava peel meal based diet. Int J Recent Res Appl Stud.

[44] Qaid MM, Al-Mufarrej SI, Azzam MM, Al-Garadi MA, Albaadani HH, Alhidary IA, et al. (2021). Growth performance, serum biochemical indices, duodenal histomorphology, and cecal microbiota of broiler chickens fed diets supplemented with cinnamon bark powder at prestarter and starter phases.

[45] Pertiwi H (2023). Detrimental effect of tannin on growth performance, viscera weight and blood biochemistry in broiler chickens reared under tropical area. Arch Razi Inst.

[46] Aro SO, Aletor VA (2012). Proximate composition and amino acids profile of different cassava tuber wastes collected from a cassava starch producing factory in Nigeria. Livest Res Rural Dev.

[47] Bona L, Van Staaveren N, Pokharel BB, Van Krimpen M, Harlander-Matauschek A (2018). The effect of low protein energy-rich diets on plasma hepatic markers, hepatic damage, and discrimination reversal learning in young female chicks. Front Vet Sci.

[48] El-Kalla F, Mansour L, Kobtan A, Elzeftawy A, Abo Ali L, Abd-Elsalam S (2018). Blood ammonia level correlates with severity of cirrhotic portal hypertensive gastropathy. Gastroenterol Res Pract.

[49] George ES, Sood S, Daly RM, Tan SY (2022). Is there an association between non-alcoholic fatty liver disease and cognitive function? A systematic review. BMC Geriatr.

[50] Senanayake SSHMML, Ranasinghe JGS, Waduge R, Nizanantha K, Alexander PABD (2015). Changes in the serum enzyme levels and liver lesions of broiler birds reared under different management conditions. Trop Agric Res.

[51] Soyinka OO, Boafo FO (2015). Growth performance, haematology and biochemical characteristics of *Clarias gariepinus* (Burchell, 1822) juveniles fed quail eggshells as replacement for dicalcium phosphate. Nig J Fish Aquacult.

[52] Abdulazeez H, Adamu SB, Igwebuike JU, Gwayo GJ, Muhammad AI (2016). Haematology and serum biochemistry of broiler chickens fed graded levels of *baobab*
*Adansonia digitata* L.) seed meal. J Agric Vet Sci.

[53] Fan Z, Chen T, Cai G, Huang X, Zhong S, Li X, et al. (2023). Effect of *Aspergillus niger* fermentation on the metabolites in corn stalks. Fermentation.

[54] Zabel RA, Morrell JJ (2020). Chapter five—fungal metabolism in relation to wood decay. Wood microbiology 2nd edition.

[55] Nick D. (1994). Nutrient requirements of poultry: ninth Revised Edition. J Appl Poultry Res.

[56] Fitzgerald F (2019). Chicken serum albumin – Fitzgerald Industries International. https://www.fitzgerald-fii.com/chicken-serum-albumin-30r-3299.html.

[57] Hashash JG, Koutroumpakis F, Anderson AM, Rivers CR, Hosni M, Koutroubakis IE, et al. (2022). Elevated serum globulin fraction as a biomarker of multiyear disease severity in inflammatory bowel disease. Ann Gastroenterol.

[58] Shuvo AAS, Rahman MS, Al-Mamum M, Islam KMS (2022). Cholesterol reduction and feed efficiency enhancement in broiler through the inclusion of nutritionally improved fermented rice bran. J Appl Poult Res.

[59] Zhai J, Wang B, Sun Y, Yang J, Zhou J, Wang T (2023). Effects of *Aspergillus niger* on cyanogenic glycosides removal and fermentation qualities of ratooning sorghum. Front Microbiol.

[60] Onodu BC, Culas RJ, Nwose EU (2018). Facts about dietary fibre in cassava: implication for diabetes’ medical nutrition therapy. Integr Food Nutr Metab.

[61] Naumann S, Schweiggert-Weisz U, Eglmeier J, Haller D, Eisner P (2019). In *vitro* interactions of dietary fibre enriched food ingredients with primary and secondary bile acids. Nutrients.

[62] Timilsena YP, Phosanam A, Stockmann R (2023). Perspectives on saponins: food functionality and applications. Int J Mol Sci.

[63] Egbune EO, Tonukari NJ (2023). Fermented mixture of cassava roots and palm kernel cake can substitute for maize in poultry feed formulation. Afr J Biochem Res.

[64] Kaiser JC, Reider H, Pabilonia KL, Moore AR (2022). Establishment of biochemical reference values for backyard chickens in Colorado (*Gallus gallus* domesticus). Vet Clin Pathol.

